# The International Medical Congress

**Published:** 1886-10

**Authors:** 


### EDITORIAL.

### The International Medical Congress.

The *London Lancet*, the most widely read and influential medical journal in the world, speaks thus cheerfully of the International Medical Congress, in its issue of July 24th :

" Our latest information is to the effect that the arrangements for the great International Congress at Washington are progressing favorably. In the case of many of our European brethren the occasion of a visit to the United States will be an unique one in their lives. We have not yet reached that familiarity with the Atlantic which is such an attainment in our American brothers,

> Qui siccis oculis monstra natantia,
> Qui vidit mare turgidum.

Nevertheless, many on this side are anxious to return the visits so generously made from the other. And whatever the discomforts of the voyage or the severity of the *mal de mer*, we are likely to have the advantage of much brotherly assistance and advice. It may mark a new era in the treatment of sea-sickness, when so many zealous physicians and surgeons are set in competition for their own relief. Be this as it may, great preparations are being made in Washington and elsewhere, and it only remains for Europe to see that the guests are forthcoming. It is the great element in all such gatherings that they be well " furnished with guests," and we would now urge on the profession and its leaders that they will do a great service by an early decision to attend, and still more by an early intimation of it to those concerned. There are American physicians who have visited England annually for thirty or forty years, and on rare occasions perhaps more than once in the year. The late Dr. Flint, whose absence will be acutely felt at Brighton this year, and at Washington next, has come of late years to think nothing so refreshing as a run to the old home of his forefathers. Let us reciprocate the compliment on this high occasion, and make the very Atlantic the measure of our desire to cultivate international science and friendship.

